# SHP2 deficiency promotes *Staphylococcus aureus* pneumonia following influenza infection

**DOI:** 10.1111/cpr.12721

**Published:** 2019-11-29

**Authors:** Wei Ouyang, Chao Liu, Ying Pan, Yu Han, Liping Yang, Jingyan Xia, Feng Xu

**Affiliations:** ^1^ Department of Infectious Diseases The Second Affiliated Hospital Zhejiang University School of Medicine Hangzhou China; ^2^ Department of Radiation Oncology The Second Affiliated Hospital Zhejiang University School of Medicine Hangzhou China

**Keywords:** chemo‐attractants, inflammatory response, macrophage polarization, protein‐tyrosine phosphatase SHP2, secondary bacterial pneumonia, type I interferon

## Abstract

**Objectives:**

Secondary bacterial pneumonia is common following influenza infection. However, it remains unclear about the underlying molecular mechanisms.

**Materials and methods:**

We established a mouse model of post‐influenza *S aureus* pneumonia using conditional *Shp2* knockout mice (*LysM^Cre/+^:Shp2^flox/flox^*). The survival, bacterial clearance, pulmonary histology, phenotype of macrophages, and expression of type I interferons and chemokines were assessed between SHP2 deletion and control mice (*Shp2^flox/flox^*). We infused additional KC and MIP‐2 to examine the reconstitution of antibacterial immune response in *LysM^Cre/+^:Shp2^flox/flox^* mice. The effect of SHP2 on signal molecules including MAPKs (JNK, p38 and Erk1/2), NF‐κB p65 and IRF3 was further detected.

**Results:**

*LysM^Cre/+^:Shp2^flox/flox^* mice displayed impaired antibacterial immunity and high mortality compared with control mice in post‐influenza *S aureus* pneumonia. The attenuated antibacterial ability was associated with the induction of type I interferon and suppression of chemo‐attractants KC and MIP‐2, which reduced the infiltration of neutrophils into the lung upon secondary bacterial invasion. In additional, *Shp2* knockout mice displayed enhanced polarization to alternatively activated macrophages (M2 phenotype). Further in *vitro* analyses consistently demonstrated that SHP2‐deficient macrophages were skewed towards an M2 phenotype and had a decreased antibacterial capacity. Moreover, SHP2 modulated the inflammatory response to secondary bacterial infection via interfering with NF‐κB and IRF3 signalling in macrophages.

**Conclusions:**

Our findings reveal that the SHP2 expression enhances the host immune response and prompts bacterial clearance in post‐influenza *S aureus* pneumonia.

## 1 INTRODUCTION

Influenza pneumonia is a significant cause of morbidity and mortality throughout the world.^1^, ^2^ There were nearly 40‐50 million deaths during the 1918 influenza pandemic and approximately 200 thousand respiratory deaths in the 2009 pandemic.^3^, ^4^ One of main reasons for most severe illness or death following influenza infection has been attributed to secondary bacterial pneumonia commonly caused by *Staphylococcus aureus* (*S aureus*), *Haemophilus influenza* and *Streptococcus pneumonia.* 5, 6 In particular*, S aureus*, including methicillin‐resistant strains, became the predominant superinfecting pathogens and caused fulminant pneumonia with fatal clinical outcomes following influenza infection.^5^, ^7^ It has been reported that the pulmonary host innate defence against secondary bacterial infection can be impaired by the preceding influenza challenge through multiple mechanisms.^8^, ^9^ Viral infection was shown to suppress function of neutrophils, leading to impaired phagocytosis and the attenuated generation of intracellular reactive oxygen species in response to subsequent bacterial infection.^6^, ^10^, ^11^ Type I interferons (IFNs), including IFN‐α and IFN‐β, were typically induced during viral infection and played an indispensable role in host defence.^12^ However, type I IFNs have been reported to facilitate post‐influenza bacterial infection by reducing the production of chemo‐attractants such as keratinocyte‐derived chemokine (KC) and macrophage inflammatory protein (MIP)‐2, which are critical for recruiting neutrophils to the site of infection.^13^, ^14^, ^15^ A recent study showed signal transducer of activation and transcription (STAT) 1, a vital transcription factor in IFN signalling, was detrimental during influenza and MRSA superinfection by suppressing type 17 immune response. STAT1 deletion resulted in increased neutrophils in lungs and decreased bacterial burden upon superinfection.^16^

In addition to neutrophils, macrophages also participate in host defence by phagocytosing and killing invasive bacteria in the lungs.^17^, ^18^ Macrophages can be phenotypically polarized towards classically activated macrophages (M1 macrophages) and alternatively activated macrophages (M2 macrophages), both of which are involved in a variety of inflammatory diseases.^19^ M1 macrophages produce high levels of pro‐inflammatory cytokines (eg, interleukin [IL]‐6, tumour necrosis factor [TNF]‐α and nitric oxide [NO]), which are important inflammatory mediators required for bacterial clearance. M2 macrophages express high levels of arginase (Arg) 1, chitinase 3‐like protein 3 (Chi3l3, Ym1) and resistin‐like α (Retnla, Fizz1), which suppress inflammation and facilitate tissue repair.^20^ Thus, upon bacterial invasion, the different phenotypes of macrophage activation determine the outcome of infection to some extent.

Src homology 2 domain‐containing phosphatase (SHP2) is a member of non‐receptor protein‐tyrosine phosphatases subfamily and ubiquitously expressed in the cytoplasm.^21^ SHP2 has been shown to regulate cellular functions in multiple lung diseases, including carcinoma, pulmonary fibrosis, chronic airway diseases and lung infection.^22^, ^23^, ^24^, ^25^ SHP2 can be activated by respiratory syncytial virus (RSV) and contributed to host antiviral activity.^26^ It also has been identified to be critical for clearance of *Haemophilus influenzae* by skewing macrophage phenotypic differentiation.^25^ In addition, SHP2 deficiency in myeloid cells alleviated pulmonary inflammation in acute lung injury.^27^ Moreover, SHP2 was found to disrupt IL‐10‐STAT3 signalling and its dependent anti‐inflammatory response in human and mouse macrophages in the context of colonic inflammation.^28^ However, to date, it remains unclear whether SHP2 is associated with susceptibility to the post‐influenza bacterial infection.

In the present study, we established a murine model of post‐influenza *S aureus* pneumonia to investigate the mechanisms involved in the impaired host antibacterial response following primary influenza challenge. Here, we demonstrat that mice with SHP2 deficiency are more susceptible to secondary *S aureus* infection. Moreover, such enhanced susceptibility is associated with the overproduction of type I IFNs and M2‐biased macrophage differentiation.

## 2 MATERIALS AND METHODS

### 2.1 Mice

*Shp2^flox/flox^* and *LysM^Cre/+^* mice on the C57BL/6 background were crossed with each other to generate conditional *Shp2* knockout mice as previously described.^23^, ^29^ *LysM^Cre/+^:Shp2^flox/flox^* mice were designated as *LysMCre:Shp2^fl/fl^,* and controls (*Shp2^flox/flox^*) were designated as *Shp2^fl/fl^* in this study. Pathogen‐free C57BL/6 mice were purchased from the Animal Center of Slaccas (Shanghai, China). All animal experiments were approved by Zhejiang University Institutional Animal Care and Use Committee (Hangzhou, China).

### 2.2 Establishment of a mouse model of post‐influenza *S aureus* pneumonia

The influenza virus PR8 strain was propagated in Madine Darby canine kidney (MDCK) cells and stored in aliquots at −80°C. Virus titers were determined using plaque assay on MDCK cells. In specific, 200 μL of the viral stock was serially diluted and incubated on MDCK monolayers at 37°C for 2 hours. After the incubation, cells were overlaid with viral growth medium (including MEM, NaHCO_3,_ 10% BSA, 1% DEAE Dextran, 1 μg/mL TPCK trypsin and 2% agarose) as described before and incubated for 72 hours at 34°C in a 5% CO_2_ atmosphere.^13^ The cells were fixed by 4% formaldehyde and stained with 1% (wt/vol) crystal violet to determine virus titers by counting the number of plaques. The wells containing of 30‐100 plaques were suitable for counting, and the virus titers was calculated by the following formula: virus titers (plaque‐forming units [PFU]/mL) = plaques × dilution × 5. The clinically isolated *S aureus* strain presenting multilocus sequence type ST15 and agr type II was cultured and counted as previously described.^30^, ^31^ In brief, *S aureus* was grown in Tryptone Soya Broth (TSB) at 37°C with shaking (200 rpm) until the log phase. The concentration of bacteria was quantified by measuring the absorbance at 600 nm according to the bacterial growth curve.

Mice (8‐10 weeks old) were intranasally inoculated with 200 PFU of the PR8 strain in 20 μL phosphate buffer solution (PBS) or PBS alone. Then, mice were subsequently intratracheally instilled with 50 μL of *S aureus* (5 × 10^7^ colony‐forming units [CFU]) or PBS 5 days after the viral infection. Mouse survival was monitored after secondary bacterial (1 × 10^7^ CFU) infection. For the KC and MIP‐2 reconstitution experiment, influenza‐infected mice were instilled with 50 μL PBS or a single dose of KC and MIP‐2 (1 μg each, R&D systems) in sterile PBS, concurrently administered with *S aureus*.

### 2.3 Quantification of PR8 virus in the lungs

The mice were sacrificed at 5 days after influenza infection. The whole lung was removed and homogenized in 1 mL PBS by mechanical grinding. After three cycles of freeze/thaw to release the virus, the supernatants of lung homogenates were collected for viral titration by plaque assay mentioned above.

### 2.4 Bacterial load in the lungs

The mice were sacrificed at indicated time points after secondary *S aureus* infection. The whole lung was homogenized in 1 mL of PBS. Bronchoalveolar lavage fluid (BALF) was collected by rinsing the lungs through a tracheal cannula with 1 mL sterile PBS three times, with about 70% of lavage fluid retrieved. Twenty μL of the fluid or homogenates was then 10‐fold serially diluted in PBS from 10^−1^ to 10^−8^. Five μL of diluted sample was plated on TSB agar plates for 24 hours incubation at 37°C. The quantification of bacteria was determined by counting the number of colonies.

### 2.5 Cell counting in BALF

BALF was collected and centrifuged for 10 min at 300 g. The supernatants were stored at −80°C until detection, and the erythrocytes of the cell pellets were removed using lysis buffer (STEMCELL, Vancouver, Canada). After total cell counting, approximately 2 × 10^5^ cells were loaded onto a slide by cytospin and stained with Giemsa stain to count the number of neutrophils.

### 2.6 Histopathological examination

The whole lung was fixed in a 4% paraformaldehyde neutral buffer solution and embedded with paraffin. The samples were sliced into 4 μm sections for haematoxylin and eosin (H&E) staining. Morphometric analysis was conducted under an optical photomicroscope (Olympus). Lung injury in the specimen was evaluated blindly and graded from 0 (normal) to 4 (severe) following four items: interstitial inflammation, neutrophil infiltration, congestion and oedema. The score was calculated by adding the individual scores for each item. Lung injury score of each mouse was calculated as the mean of four lung sections.^32^

### 2.7 Cell culture and in vitro experiments

Primary mouse peritoneal macrophages (PMs) from *Shp2^fl/fl^* and *LysMCre:Shp2^fl/fl^* mice were elicited using thioglycolate broth (Sigma‐Aldrich) as previously described.^33^, ^34^ The cells were seeded in six‐well plates and cultured in RPMI 1640 medium containing 10% fetal bovine serum at 37°C in a 5% CO_2_ atmosphere. After 4 hours cultivation, non‐adherent cells were removed. A total of 10^6^ adherent PMs were stimulated with polyinosinic and polycytidylic acid (poly[I:C]), a synthetic analog of dsRNA (InvivoGen, San Diego, CA, USA), for 24 hours and subsequently inoculated with *S aureus* (multiplicity of infection [MOI], 10 CFU per cell) for the indicated time points. The cells were collected for mRNA or protein detection. For the IFN‐α pre‐treatment experiment, recombinant IFN‐α (1000 U/mL) (R&D systems, Minneapolis, MN, USA) was added to the cell culture 1 hour prior to 6 hours incubation with *S aureus* (MOI, 10). After stimulation, the supernatants were collected and stored at −80°C for cytokine measurement.

### 2.8 Enzyme‐linked immunosorbent assay (ELISA)

Cell‐free BAL fluid or cell culture supernatants were applied for ELISA kits to determine levels of myeloperoxidase (MPO) (Cloud‐Clone Corp), IFN‐α, IFN‐β (InvivoGen), KC and MIP‐2 (Multiscience) according to manufacturers' instructions.

### 2.9 Quantitative real‐time PCR (qPCR)

Total RNA from PMs or the whole lung of mice was extracted using an Ultrapure RNA kit (cwbiotech). Then, 1 μg of total RNA (DNase‐treated) was reverse‐transcribed into cDNA with HiFiScript cDNA synthesis kit (cwbiotech). The reaction of reverse transcriptase PCR was performed on 2720 Thermal Cycler (Applied Biosystems). Each sample has been excluded the existence of endogenous genomic DNA without reverse transcriptase as control. The 1 μL cDNA products were used as template for qPCR amplification using SYBR Green PCR Master Mix (cwbiotech) in the CFX96 Touch Real‐Time PCR Detection System (Bio‐Rad). The primer sequences are shown in Table 1. The relative expression of the target genes using the calculated threshold cycle (Ct) was normalized with β‐actin, and fold change compared with control was determined using the ΔΔCt method.

**Table 1:** Primer sequences for qPCR detection

Gene	Forward primers (5′‐3′)	Reverse primers (5′‐3′)
β‐actin	GTATCCTGACCCTGAAGTACC	GAAGGTCTCAAACATGATCT
Shp2	GAAACGGTCATTCAGCCACT	GCAGCCAAGGAGTCATCTTC
IL‐6	AGTTGCCTTCTTGGGACTGA	TCCACGATTTCCCAGAGAAC
TNF‐α	CTGGGACAGTGACCTGGACT	GCACCTCAGGGAAGAGTCTG
IL‐12b	GTCCTCAGAAGCTAACCATCTCC	CCAGAGCCTATGACTCCATGTC
iNOS	GTTCTCAGCCCAACAATACAAGA	GTGGACGGGTCGATGTCAC
Arg‐1	CAGAAGAATGGAAGAGTCAG	CAGATATGCAGGGAGTCACC
Ym‐1	GGATGGCTACACTGGAGAAA	AGAAGGGTCACTCAGGATAA
Fizz1	CCCTCCACTGTAACGAAG	GTGGTCCAGTCAACGAGTAA
IFN‐α	TACTCAGCAGACCTTGAACCT	CAGTCTTGGCAGCAAGTTGAC
IFN‐β	ATGAGTGGTGGTTGCAGGC	TGACCTTTCAAATGCAGTAGATTCA

### 2.10 Flow cytometry

A single‐cell suspension was derived from the BALF of *LysMCre:Shp2^fl/fl^* and *Shp2^fl/fl^* mice 24 hour after secondary *S aureus* infection. After blocking the Fc receptor, cells were stained with CD11b (clone M1/70, BioLegend, San Diego), F4/80 (clone BM8, BioLegend) and Dectin‐1 (clone RH1, BioLegend), or their corresponding IgG isotype controls. After extracellular markers were stained, the cells were fixed, permeabilized and stained with CD206 (clone C068C2, BioLegend) or its isotype control. Cell samples were detected by CytoFLEX flow cytometer (Beckman Coulter, Inc.), and FlowJo software (TreeStar, Inc.) was used for analysis.

### 2.11 Phagocytosis assay

The *S aureus* was labelled to assess the phagocytic capability of macrophages.^35^ Briefly, bacteria in log phase were washed twice with PBS and labelled in 0.1 mg/mL 5(6)‐FITC (MedChemExpress, New jersey, USA) in 0.9% NaCl for 1 hour at 37°C with shaking. PMs were stimulated with poly(I:C) for 24 hour and then incubated with FITC‐labelled *S aureus* (MOI: 20) for additional 1 and 2 hours, respectively. Infected cells were collected and extensively washed with PBS, and the bacterial uptake capability of the cells was detected by flow cytometry.

### 2.12 Western blot

The protein was extracted from peritoneal macrophages using 1× RIPA lysis buffer containing 1mM phenylmethylsulfonyl fluoride and protease inhibitor cocktail. Protein concentrations were determined by the BCA Protein Assay Kit (Beyotime). The total proteins (30 μg) were separated on 10% SDS‐PAGE gel and transferred to PVDF membranes. After blocking in Tris‐buffered saline Tween‐20 with 5% fresh non‐fat milk, the target proteins on the membranes were detected overnight at 4°C with the following antibodies: β‐actin, SHP2, nuclear factor (NF)‐κB p65, p‐p65, interferon regulatory factor 3 (IRF3), p‐IRF3 and mitogen‐activated protein kinases (MAPKs) including JNK, p‐JNK, p38, p‐P38, Erk and p‐Erk (Cell Signaling Technology, Inc.).

### 2.13 Statistical analysis

All data analyses were performed using GraphPad Prism version 5 (GraphPad Software, Inc.). Quantitative data are expressed as mean ± SEM, and significance in differences was determined with student *t* test or one‐way ANOVA with Bonferroni's post hoc analysis where appropriate. Mouse survival rates were analysed with log‐rank test. A threshold of *P* < .05 was considered statistically significant.

## 3 RESULTS

### 3.1 SHP2 deficiency leads to higher mortality and impaired bacterial clearance in mice with post‐influenza *S aureus* pneumonia

**Figure 1:**
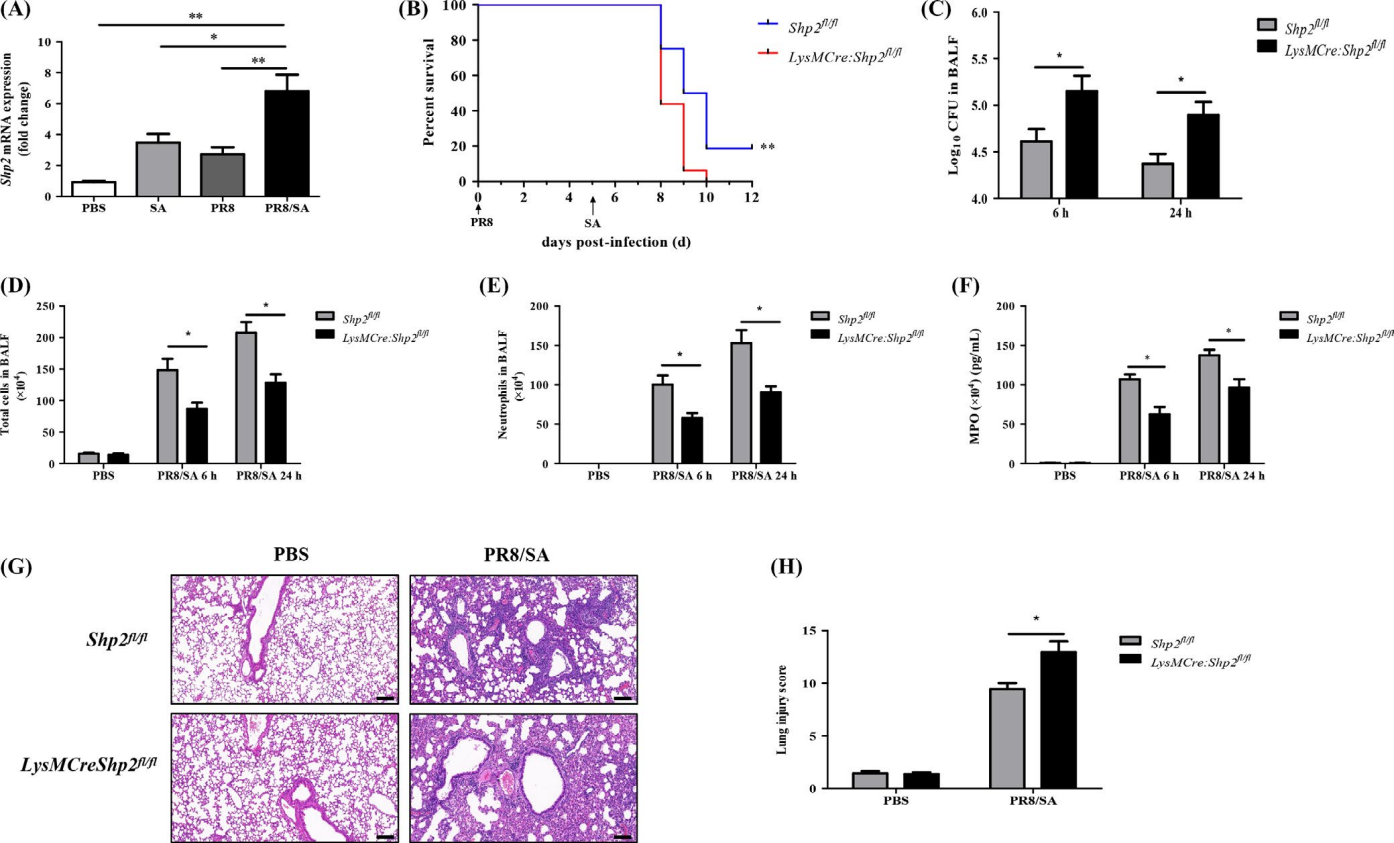
SHP2 deficiency leads to higher mortality and decreased neutrophil accumulation in mice with PR8/*S aureus* coinfection. (A) The lungs were harvested from the mice challenged with PBS/PBS, PR8 (200 PFU)/PBS, PBS/*S.aureus* (SA) (5 × 10^7^ CFU) or PR8/SA. The *Shp2* mRNA expression was detected by qPCR. n = 5 mice per group. *P < .05, **P < .01. Data are representative from 3 independent experiments. (B) Following infection of influenza PR8 (200 PFU) for 5 d, mouse survival was monitored in *Shp^fl/fl^* and *LysMCre:Shp2^fl/fl^* mice after secondary SA (1 × 10^7^ CFU) challenge. n = 16 mice per group, **P < .01. Data are combined from 2 separate experiments. (C‐H) *Shp^fl/fl^* and *LysMCre:Shp2^fl/fl^* mice were infected with 200 PFU of PR8 for 5 d and then challenged with SA (5 × 10^7^ CFU) for 6 h or 24 h. Mice treated with PBS alone were used as negative controls. The bacterial load (C) in the BALF was measured. The number of total cells (D) and neutrophils (E) was counted, and the level of MPO (F) was determined in the BALF. Representative results of lung tissues by H&E staining (G) and the histogram of lung injury scores (H) were shown 24 h after secondary SA infection. Magnification for HE sections: 100×, scale bar: 100 μm. n = 4‐6 mice per group, *P < .05. Data are representative of 2 independent experiments with similar results

SHP2 has been reported to be activated and involved in host defence against infection.^25^, ^26^ Interestingly, we found that the level of SHP2 expression was upregulated by either influenza or *S aureus* infection and was much higher in mice with post‐influenza *S aureus* pneumonia (Figure 1A and Figure S1A). Then, we challenged control (*Shp2^fl/fl^*) and *Shp2* conditional knockout mice (*LysMCre:Shp2^fl/fl^*) with influenza PR8 or *S aureus* alone. There was no obvious difference in viral or bacterial burden in lung between control and *Shp2* knockout mice (Figure S1B,C). However, an increase in the expression of type I IFNs was observed in SHP2 deficient mice at day 5 post‐influenza infection, compared to control mice (Figure S1D,E). These data suggest SHP2 plays a limited role in either PR8 viral or *S aureus* pneumonia. To further evaluate the role of SHP2 in viral and bacterial coinfection, survival was observed between the *Shp2^fl/fl^* and *LysMCre:Shp2^fl/fl^* mice. The results showed *LysMCre:Shp2^fl/fl^* mice had a lower survival rate than *Shp2^fl/fl^* mice after secondary bacterial infection (Figure 1B). Furthermore, compared to the control mice, *LysMCre:Shp2^fl/fl^* mice displayed an increase of bacterial number in BALF during the early stages following secondary *S aureus* challenge (Figure 1C). SHP2 deletion also led to a reduction in the number of total cells and neutrophils in BALF and a decreased level of MPO after secondary bacterial infection (Figure 1D‐F). The histopathological analysis showed severe lung injury in *LysMCre:Shp2^fl/fl^* mice due to increased bacterial burden compared to control mice (Figure 1G,H). Collectively, these findings demonstrate that SHP2 deficiency leads to an attenuated recruitment of neutrophils, which could not restrict the bacterial infection effectively in the lung.

### 3.2 Deletion of SHP2 results in enhanced induction of type I IFN and attenuated production of chemokines in mice with secondary *S aureus* infection

**Figure 2:**
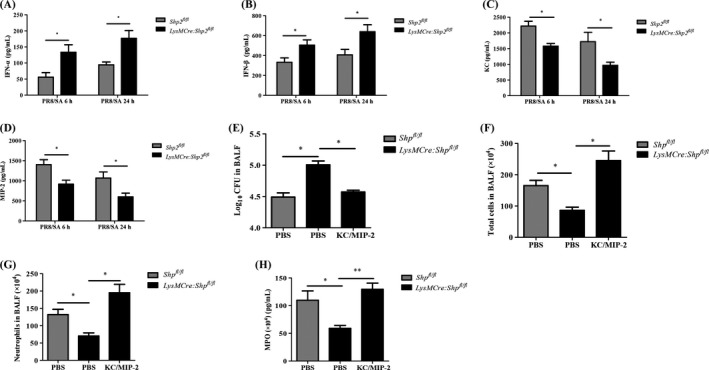
Deletion of SHP2 results in high levels of type I IFN and attenuated production of chemokines in mice with secondary *S aureus* infection. (A‐C) *Shp^fl/fl^* and *LysMCre:Shp2^fl/fl^* mice were treated with 5 × 10^7^ CFU *S aureus* (SA) for 6 h or 24 h, following an inoculation with 200 PFU PR8 for 5 d. The concentrations of IFN‐α (A), IFN‐β (B), KC (C) and MIP‐2 (D) in the BALF were detected by ELISA. n = 4 mice per group. (E‐H) Mice were challenged with PR8 (200 PFU) for 5 d and then treated by an instillation of SA (5 × 10^7^ CFU), with or without recombinant murine KC and MIP‐2 (1 μg each), for 6 h. The bacterial load (E) in the BALF was detected. The number of total cells (F) and neutrophils (G), as well as the level of MPO (H) in the BALF, was determined. n = 4 mice per group. *P < .05, **P < .01. Representative data of 2‐3 independent experiments are shown

During influenza infection, the induction of type I IFNs has been shown to be closely related to secondary bacterial pneumonia.^13^, ^14^ The current study revealed that SHP2 deletion led to a high level of IFN‐α, IFN‐β and the decreased production of chemokines such as KC and MIP‐2 in the BALF, upon secondary *S aureus* infection (Figure 2A‐D). Shahangian et al found that type I IFN‐induced susceptibility to secondary *S pneumonia* infection was attributed to decreased production of KC and MIP‐2.^13^ Similarly, IFN‐α pre‐treatment markedly abated the increased levels of KC and MIP‐2 in primary PMs from C57BL/6 mice upon *S aureus* infection (Figure S2A,B). Thus, we further examined whether an additional infusion of KC and MIP‐2 can reconstitute the antibacterial immune response in *LysMCre:Shp2^fl/fl^* mice. Following infection with influenza, *Shp2* knockout mice were treated with both *S aureus* and a dose of KC plus MIP‐2. The results showed that the exogenous administration of KC and MIP‐2 significantly reduced the secondary *S aureus* burden in the BALF of *LysMCre:Shp2^fl/fl^* mice, compared to the co‐infected counterpart mice that just received with PBS carrier (Figure 2E). The number of total cells and neutrophils was also substantially upregulated, so were the levels of MPO in *LysMCre:Shp2^fl/fl^* mice treated with KC and MIP‐2 (Figure 2F‐H). Our data highlight the critical role of KC and MIP‐2 in the clearance of post‐influenza *S aureus* infection by promoting neutrophil recruitment into the lung.

### 3.3 Deletion of SHP2 suppresses inflammatory cytokines via modulating macrophage phenotype in mice upon secondary *S aureus* infection

**Figure 3:**
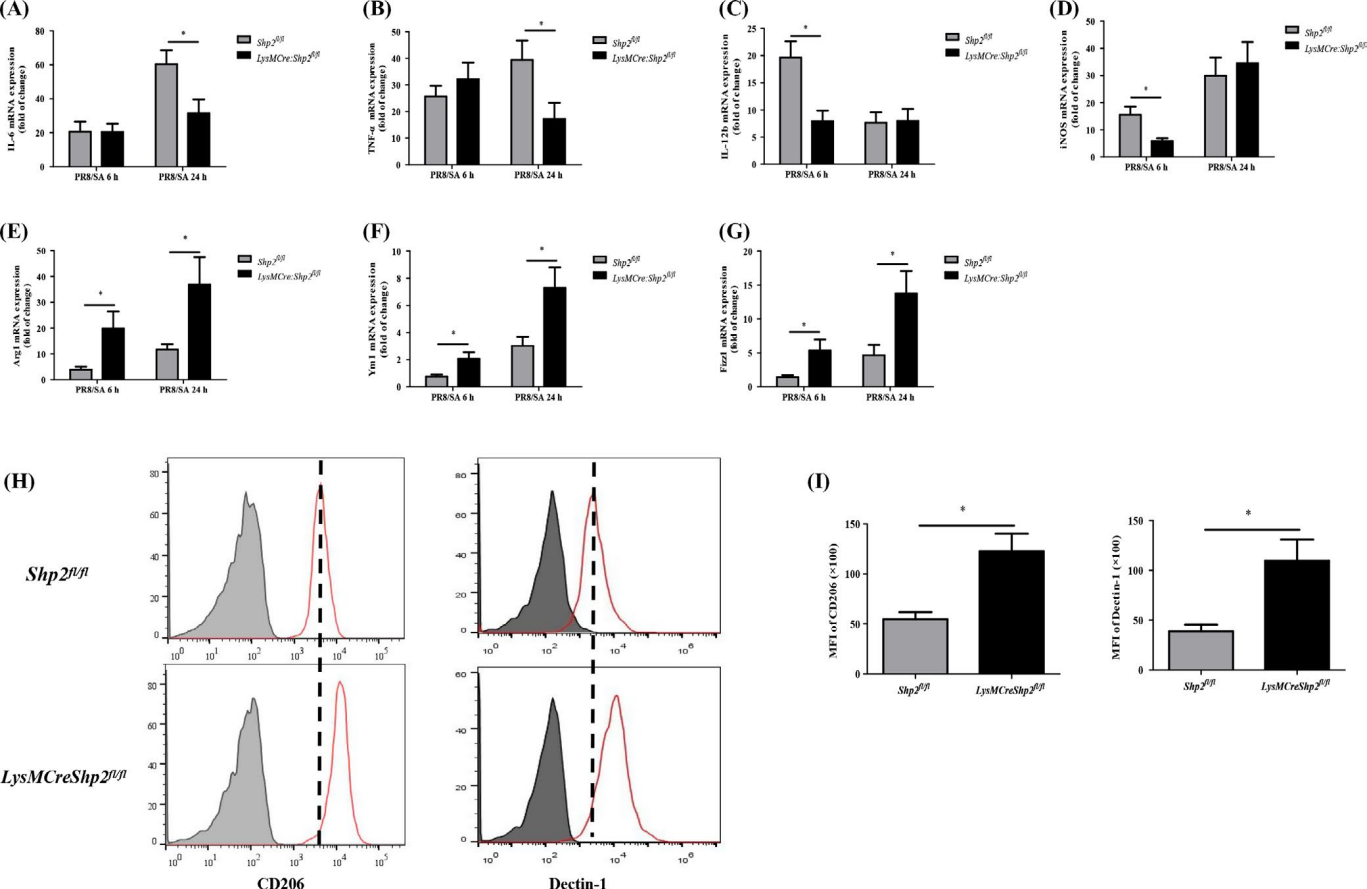
Deletion of SHP2 suppresses inflammatory cytokine production via modulation of the macrophage phenotype in mice with secondary *S aureus* infection. *Shp^fl/fl^* and *LysMCre:Shp2^fl/fl^* mice were treated with 5 × 10^7^ CFU *S aureus* (SA) at 5 d after an inoculation of 200 PFU PR8 influenza. (A‐G) The relative levels of mRNA for M1‐associated genes including IL‐6, TNF‐α, IL‐12b, iNOS and M2 genes (eg, Arg1, Ym1 and Fizz1) in lung tissues were assessed by qPCR at 6 h and 24 h after secondary SA infection. n = 5 mice in each group. *P < .05. (H‐I) CD206 and Dectin‐1 expression on CD11b^+^ F4/80^+^ macrophages from the BALF was analysed by flow cytometry, and the mean fluorescence intensity (MFI) of CD206 and dectin‐1 was calculated 24 h after secondary SA infection. n = 3 mice per group. *P < .05. Representative data of 2‐3 independent experiments are shown

In addition to neutrophils, macrophages also participate in the host immune response to post‐influenza bacterial infection. Chen et al found that M2 macrophages led to a hypersusceptibility to secondary bacterial infection.^36^ Our data showed that compared to the control mice, *Shp2* knockout mice displayed significantly lower levels of M1‐related genes (eg, IL‐6, TNF‐α, IL‐12b and inducible NO synthase [iNOS]) (Figure 3A‐D) and elevated expression of M2‐related genes including Arg1, Ym1 and Fizz1 (Figure 3E‐G), upon dual infection. Moreover, there was an upregulated expression of the M2 surface marker CD206 and Dectin‐1^37^ on CD11b^+^ F4/80^+^ macrophages from *LysMCre:Shp2^fl/fl^* mice after secondary bacterial infection, compared to *Shp2^fl/fl^* mice (Figure 3H,I). Thus, the results indicate that SHP2 deficiency reduces the expression of inflammatory genes through regulating macrophages polarization, which could pre‐dispose the mice to post‐influenza bacterial infection.

### 3.4 Loss of SHP2 skews macrophage differentiation in response to poly(I:C) and *S aureus* co‐stimulation

**Figure 4:**
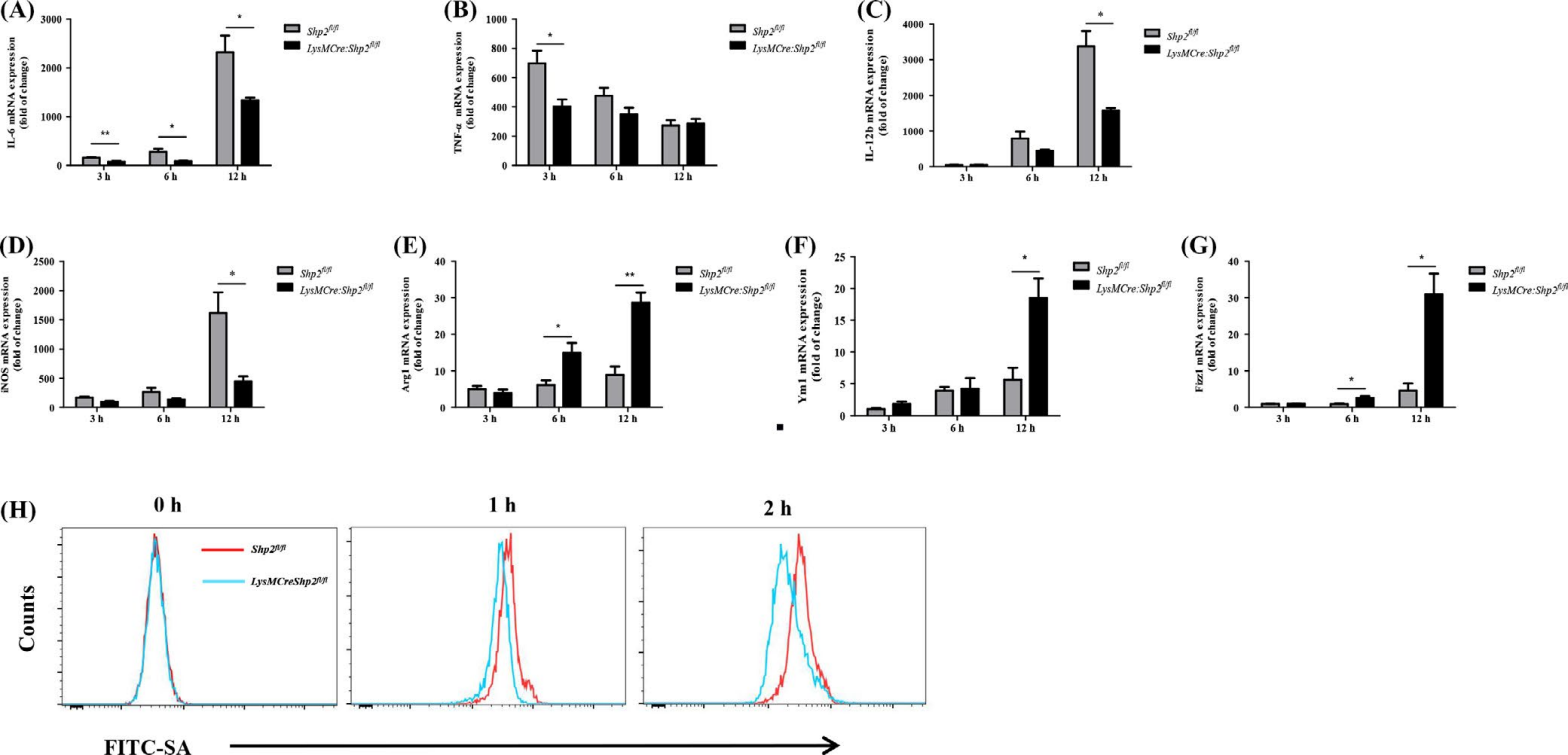
Loss of SHP2 skews macrophage differentiation to M2 phenotype and abates its phagocytic activity in response to poly(I:C) and *S aureus* dual stimulation. The peritoneal macrophages from *Shp^fl/fl^* and *LysMCre:Shp2^fl/fl^* mice were stimulated with poly(I:C) (20 μg/mL) for 24 h and then incubated with *S aureus* (MOI, 10) for 3, 6 and 12 h, respectively. (A‐D) Expression of M1 gene marker including IL‐6, TNF‐α, IL‐12b and iNOS was measured by qPCR. (E‐G) Expression of M2 gene markers (eg, Arg‐1, YM‐1 and Fizz1) was determined by qPCR. *P < .05, **P < .01. (H) After pre‐stimulation by poly(I:C) (20 μg/mL) for 24 h, the PMs were incubated with FITC‐labelled bacteria for 1 and 2 h. Then, the fluorescence positivity was analysed by flow cytometry. Representative traces of bacteria engulfed were shown by red (*Shp^fl/fl^*) and blue line (*LysMCre:Shp2^fl/fl^*), respectively. Data are representative of 3 independent experiments

The mechanisms by which SHP2 regulates the inflammation response to the dual infection were further dissected in PMs. Upon poly(I:C), a synthetic analog of viral dsRNA, and *S aureus* serial stimulation, the expression of prototypical M1 genes (eg, IL‐6, TNF‐α, IL‐12b and iNOS) was reduced (Figure 4A‐D), whereas the levels of the M2‐associated genes (eg, Arg1, Ym1 and Fizz1) were significantly increased in SHP2‐deleted PMs (Figure 4E‐G), compared to those from *Shp2^fl/fl^* mice. M1‐polarized macrophages generally displayed a constrained bactericidal activity.^38^ Therefore, we evaluated the phagocytic ability in macrophages from control and SHP2‐deficient mice. After stimulation of poly(I:C), the cells were then cultured with FITC‐labelled *S aureus*. We found the internalization of bacteria was increased in control macrophages after 2 hours infection, compared with SHP2‐deleted cells (Figure 4H). The data implicate that SHP2 deficiency not only restricts expression of pro‐inflammatory cytokines but also cripples phagocytosis capability upon the dual infection.

### 3.5 SHP2 is required for the phosphorylation of NF‐κB and IRF3 in response to poly(I:C) and *S aureus* dual stimulation

**Figure 5:**
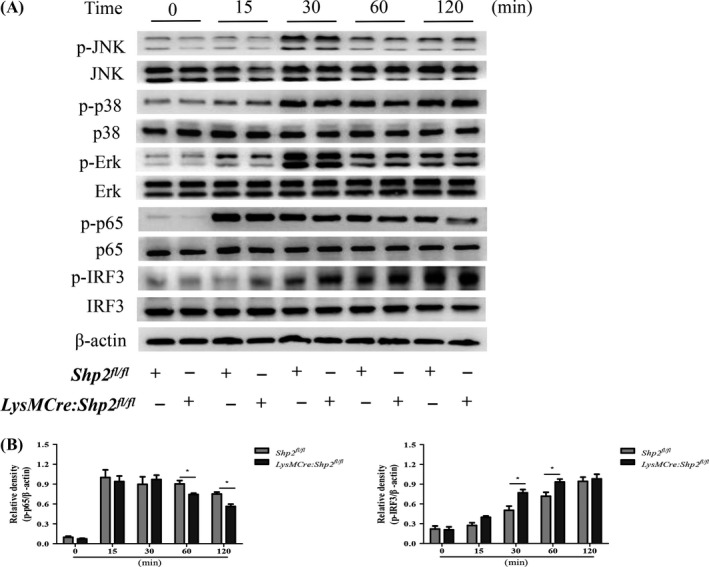
SHP2 regulates NF‐κB and IRF3 signalling upon poly(I:C) and *S aureus* serial stimulation. The peritoneal macrophages from *Shp^fl/fl^* and *LysMCre:Shp2^fl/fl^* mice were stimulated with poly(I:C) (20 μg/mL) for 24 h and then incubated with *S aureus* (MOI, 10) for 15, 30, 60 and 120 min, respectively. Phosphorylated and total protein levels of JNK, p38, Erk1/2, p65 and IRF3 were detected by Western blot. Representative images (A) and quantitative analysis of blots (B) from 3 independent experiments were shown. *P < .05

It has been well established that NF‐κB p65 can activate inflammatory cytokines and induce M1‐related gene expression,^39^ while IRF‐3 is a central regulatory molecule responsible for activating expression of type I IFNs.^40^ To illuminate the underlying molecular mechanisms for the SHP2‐mediated inflammatory response during viral and bacterial serial infection, the main inflammatory signalling pathways were detected in vitro. The data revealed that dual stimulation (poly[I:C] and *S aureus*) induced the phosphorylation of MAPKs (JNK, p38 and Erk1/2), NF‐κB p65 and IRF3 in PMs. Although SHP2 deletion had no effect on the increased phosphorylation of MAPKs, it caused a remarkable decrease of phosphorylated NF‐κB p65 at 60 and 120 minutes. Also, it caused an apparent increase of phosphorylated IRF3 at 30 and 60 minutes after dual stimulation (Figure 5A,B). Therefore, SHP2 holds a discrepancy role in regulating NF‐κB‐dependent inflammatory cytokines expression and IRF3‐dependent interferon production.

## 4 DISCUSSION

Secondary bacterial pneumonia following influenza infection is often severe with high mortality. In 1979, Jakab et al reported that influenza A increased susceptibility to *S aureus* infection.^41^ In the 2009 H1N1 pandemic, the aetiological importance of *S aureus* as a cause of post‐influenza pneumonia had been further recognized among fatal cases.^42^, ^43^ However, the underlying molecular mechanisms for secondary bacterial pneumonia remain to be further illustrated. In the present study, we demonstrate that tyrosine phosphatase SHP2 is involved in the immune response to post‐influenza *S aureus* pneumonia. Moreover, mice with myeloid‐restricted ablation of SHP2 show more susceptible to secondary bacterial infection due to the overproduction of type I IFNs and M2‐biased macrophage differentiation.

Type I IFNs are elicited as part of the host immune response to influenza virus infection. Loss of type I IFN led to exacerbated disease pathology characterized by pulmonary infiltration of granulocytes.^44^ However, antiviral IFN pathways may potentiate secondary bacterial infection. It has been reported that influenza‐induced type I IFN inhibited Th17 immunity and increased susceptibility to secondary *S aureus* pneumonia.^45^ Nakamura et al reported that an enhanced type I IFN response was associated with decreased production of the chemokine CCL2, which impaired the recruitment of macrophages and bacterial clearance in mice co‐infected with influenza virus and *S pneumonia.* 46 Moreover, other studies have demonstrated that type I IFNs induced by viral infection led to insufficient elimination of bacteria by inhibiting the production of chemokines (eg, KC and MIP‐2) responsible for neutrophil recruitment into the lungs.^13^, ^15^ Conversely, the overexpression of KC in the transgenic mice displayed a beneficial effect on bacterial clearance, which was related to a vigorous migration of neutrophils in the lung.^47^ Interestingly, intratracheal inoculation of KC and MIP‐2 could effectively recover the host resistance to post‐influenza *S pneumonia* infection in IFN receptor knockout mice, by restoring the neutrophil response during the early course of pulmonary infection.^13^ However, a recent study showed type I IFNs contributed to the enhanced MRSA clearance in IL‐21 receptor knockout mice via inducing granzyme B by neutrophils and anti‐IFNAR1 treatment diminished MRSA clearance.^48^ Our data showed that the deletion of SHP2 increased the production of type I IFNs which in turn inhibited the expression of KC and MIP‐2, resulting in an insufficient recruitment of neutrophils to the lungs upon secondary bacterial challenge. Exogenous administration of KC and MIP‐2 effectively reconstituted the impaired host defence to post‐influenza bacterial infection in *LysMCre:Shp2^fl/fl^* mice, confirming that chemokine‐mediated recruitment of neutrophils to the site of infection was one of the key mechanisms involved in antibacterial innate immunity.

Macrophages exhibit remarkable plasticity during the maturation process and can be differentiated into either M1 or M2 phenotype. M1 macrophages produce abundant pro‐inflammatory mediators and promote bactericidal activity, whereas M2 macrophages are associated with the resolution of inflammation and persistence of bacteria.^49^ A previous study reported that M2 macrophages were in an activated state during influenza infection and induced an impaired host innate immune response at the early antibacterial process.^36^ Gopal et al claimed that STAT2 deficiency increased accumulation of M1, M2 and M1/M2 co‐expressing macrophages by influenza‐MRSA superinfection, which was associated with increased bacterial clearance.^50^ SHP2‐mediated regulation in macrophages has been shown to restrain the IL‐4‐induced M2 phenotype in pulmonary fibrosis.^23^ The antibacterial M1 macrophages in *Haemophilus influenzae* pneumonia were preferentially activated via SHP2‐dependent NF‐κB p65 signalling.^25^ In addition, disruption of SHP2 in monocyte/macrophages was found to alleviate neutrophil recruitment and inflammation in LPS‐induced acute lung injury,^27^ and conditional knockout of *Shp2* in the lung epithelia was shown to reduce pulmonary inflammation in cigarette smoke‐exposed mice.^24^ These studies supported the role of SHP2 for driving macrophages towards an M1 phenotype and enhancing the inflammatory response. In contrast, Guo et al reported that SHP2 negatively regulated NLRP3 activation and decrease overproduction of pro‐inflammatory cytokines including IL‐1β and IL‐18 in macrophages.^51^ Therefore, the role of SHP2 in the innate immunity appears to be diverse dependent on different immune cells and disease models. In the current study, we revealed that SHP2 deletion increased the expression of M2‐associated markers and decreased the levels of M1 markers in both primary macrophages and the lungs upon secondary bacterial infection. And the altered macrophage phenotype induced by SHP2 deficiency contributed to poor host antibacterial immunity in the coinfection model.

NF‐κB is activated in response to various stimuli or stresses. NF‐κB p65 was reported to regulate the expression of the pro‐inflammatory and antibacterial genes in macrophages.^52^, ^53^, ^54^ We previously demonstrated that infection with *S aureus* induced phosphorylation of NF‐κB p65 in macrophages, which shifted the cells into M1 phenotype and promoted the antibacterial response.^38^ In this study, we further revealed that SHP2 could drive M1 macrophage polarization and amplify the inflammatory response by activation of NF‐κB p65 in response to dual stimulation. IRF3, a member of IRF family, is the key transcription factor involved in the activation of IFN‐α/β expression.^55^ An et al reported that SHP2 negatively regulated TLR3/TLR4 induced TIR‐domain‐containing adapter‐inducing IFN‐β (TRIF)‐dependent type I IFN production.^34^ In addition, Park et al recently identified SHP2 as a negative regulator of TLR2‐induced IFN‐β production.^56^ Consistent with these findings, our data demonstrated IRF3 to be further activated upon dual stimulation with poly(I:C) and *S aureus* in *vitro*, which could be responsible for the increased IFN production in SHP2‐deficient macrophages.

In summary, this study dissects the molecular mechanisms associated with post‐influenza bacterial infection. We identify SHP2 as one of key factors required to mount a vigorous immune response to a secondary bacterial infection. Moreover, SHP2 regulates negatively the production of type I IFNs and induces the M1‐biased macrophage differentiation required for a protective antibacterial inflammatory response. Our findings highlight the importance of innate immunity for restricting secondary bacterial infection in the lungs following influenza infection.

## CONFLICT OF INTEREST

The authors declare they have no conflict of interest.

## AUTHOR CONTRIBUTIONS

F Xu and JY Xia originated the hypothesis and designed the experiments; W Ouyang, C Liu and Y Pan performed experiments; W Ouyang, Y Han and LP Yang carried out the data analyses; W Ouyang and F Xu wrote the manuscript.

## Data Availability

The data that support the findings of this study are available from the corresponding author upon reasonable request.
