# Supplementary figures and images for: SHP2 deficiency promotes Staphylococcus aureus pneumonia following influenza infection
Source: Cell Prolif. 2019 Nov 29;53(1):e12721. doi: 10.1111/cpr.12721 (PMC6985656; doi:10.1111/cpr.12721)

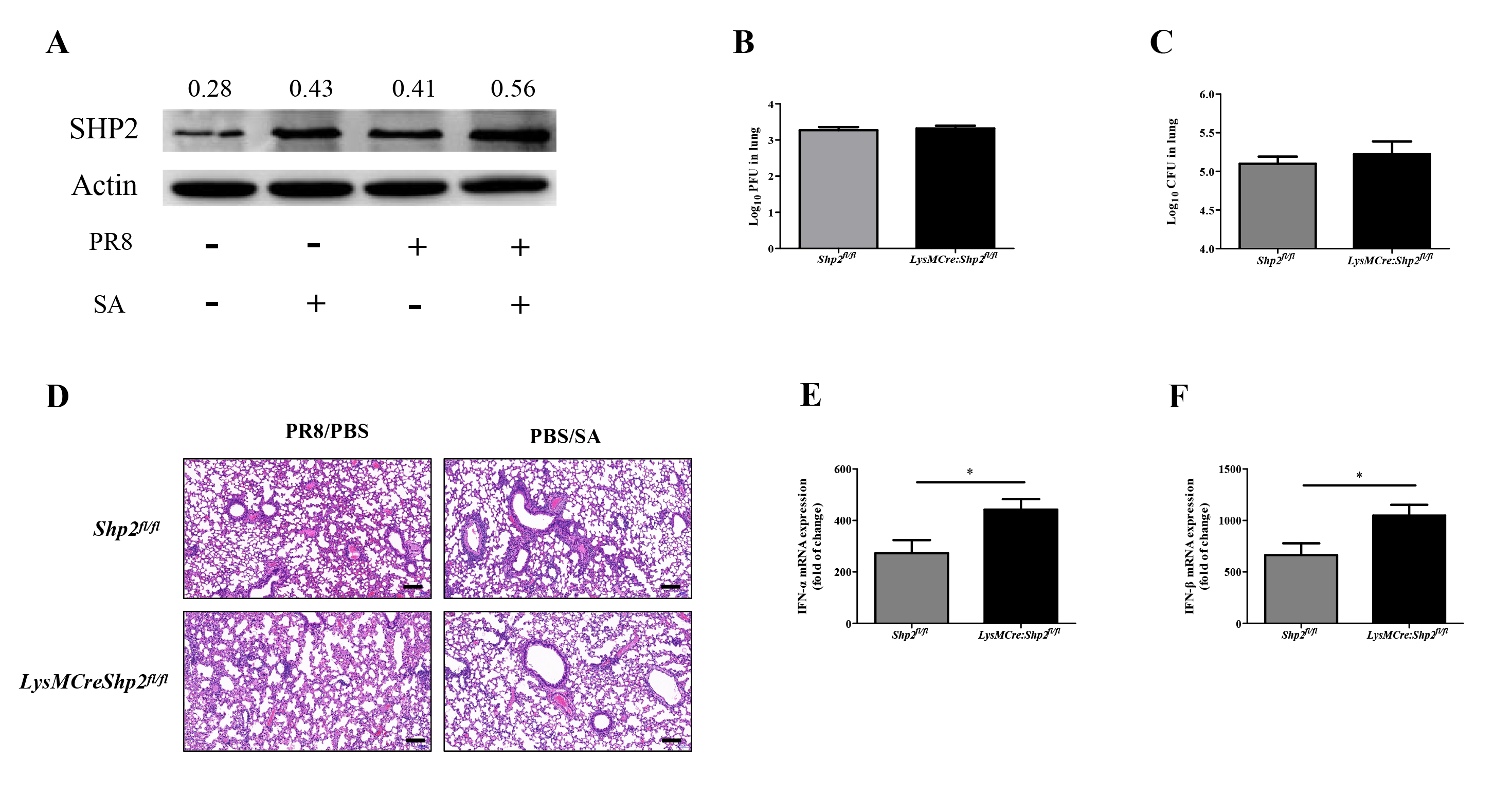

Supplement: Supplementary file 1 [file CPR-53-e12721-s001.tif]

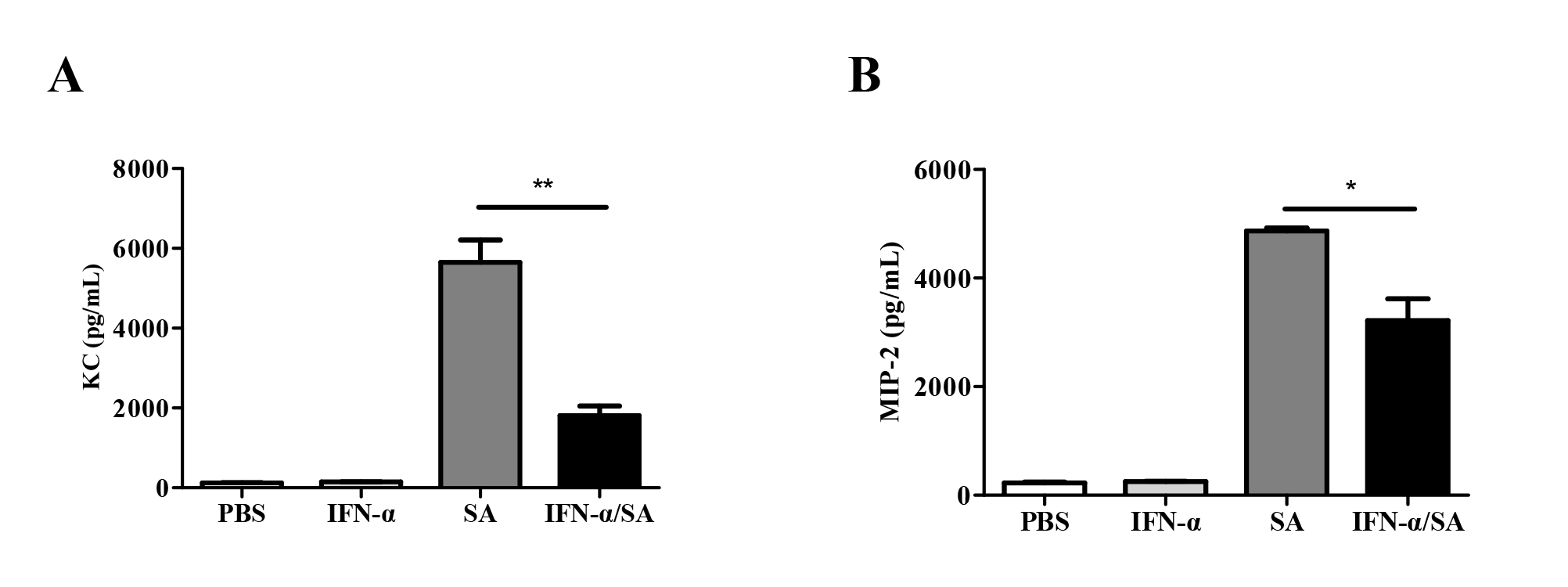

Supplement: Supplementary file 2 [file CPR-53-e12721-s002.tif]
