# Supplementary material for: SHP2 deficiency promotes Staphylococcus aureus pneumonia following influenza infection
Source: Cell Prolif. 2019 Nov 29;53(1):e12721. doi: 10.1111/cpr.12721 (PMC6985656; doi:10.1111/cpr.12721)
Supplement: Supplementary file 3 [file CPR-53-e12721-s003.docx]

SHP2 deficiency promotes *Staphylococcus aureus* pneumonia following influenza infection

Wei Ouyang ^1^, Chao Liu ^1^, Ying Pan ^1^, Yu Han ^1^, Liping Yang^1^, Jingyan Xia ^2^, Feng Xu ^1^

1 Department of Infectious Diseases, The Second Affiliated Hospital, Zhejiang University School of Medicine, Hangzhou, 310009, China

2 Department of Radiation Oncology, The Second Affiliated Hospital, Zhejiang University School of Medicine, Hangzhou, 310009, China

**Supplementary Figure 1: SHP2 deficiency exerts limited effects on viral and**

**bacterial clearance in mice with influenza or *S.aureus* infection.**

**(A)** The lungs were harvested from the mice challenged with PBS, PR8 (200 PFU)/PBS, PBS/SA (5 × 10^7^ CFU), and PR8/SA. SHP2 expression in lung tissues by immunoblot was shown. Numbers above the lanes indicated the relative ratio of SHP2/Actin. **(B-F)** Viral and bacterial loads in lung were examined by plaque and CFU assay, respectively (**B-C)**. *Shp2^fl/fl^* and *LysMCre:Shp2^fl/fl^* mice were challenged with PR8 influenza (200 PFU) intranasally for 5 days or *S.* *aures* (5 × 10^7^ CFU) intratracheally for 24 h. H&E stains of lung sections were examined (magnification: 100 ×, scale bar: 100 μm) **(D)**. Expression of IFN-α and IFN-β mRNA in whole lungs was detected by qPCR **(E-F)**. *n* = 4 mice in each group. **p* < 0.05. Data are representative of 2 independent experiments with similar results.

**Supplementary Figure 2: Type I IFN inhibits production of KC and MIP-2 in macrophages upon *S. aureus* stimulation.**

The peritoneal macrophages from C57BL/6 mice were pretreated with IFN-α (1000 U/mL) for 1 h prior to infection by *S. aureus* (SA) (MOI, 10) for 6 h. The levels of KC **(A)** and MIP-2 **(B)** in cell culture supernatants were detected by ELISA. **p* < 0.05, ***p* < 0.01. Representative data of 3 independent experiments are shown.
